# The importance of morphological changes in neutrophils in the diagnosis of bacterial infections in dogs with confirmed urinary tract infections in a Veterinary Care Service, Rio de Janeiro, Brazil

**DOI:** 10.29374/2527-2179.bjvm0004022

**Published:** 2023-07-24

**Authors:** Paulo Daniel Sant’Anna Leal, Ianna Barbosa Lima Veeren, Solange Fonseca, Carlos Henrique Machado, Carlos Wilson Gomes Lopes

**Affiliations:** 1 Veterinarian, DSc., Programa de Pós-Graduação em Ciências Veterinárias (PPGCV), Departamento de Parasitologia Animal (DPA), Instituto de Veterinária (IV), Universidade Federal Rural do Rio de Janeiro (UFRRJ). Seropédica, RJ, Brazil.; 2 Veterinarian, autonomous. Rua Januário José Pinto de Oliveira, 735, Recreio dos Bandeirantes, RJ, Brazil.; 3 Microbiologist, Hospital Getúlio Vargas, Penha Circular, Rio de Janeiro, RJ, Brazil.; 4 Veterinarian, DSc., Departamento de Clínica e Cirurgia Veterinária, IV, UFRRJ. Seropédica, RJ, Brazil.; 5 Veterinarian, PhD., DPA, IV, UFRRJ. Seropédica, RJ, Brazil.

**Keywords:** toxic neutrophils, bacterial infection, urine culture, microbiological diagnosis, dogs, neutrófilos tóxicos, infecção bacteriana, urocultura, diagnóstico microbiológico, cães

## Abstract

Neutrophils (PMNs) are cellular markers used for diagnosing inflammation and/or infections. In this study, the objective was to highlight the importance of recording the toxic morphological alterations of the PMNs as markers of infection in 10 cases, positive bacterial isolation by culture due to dysuria, hematuria and/or fetid urine, as manifestations of urinary tract disease. Smear observations were performed by immersion for counting and morphological evaluations of 3,000 leukocytes in smears and in leukocyte concentrate. One (10.0%) of the dogs had leukocytosis, and two (20.0%) dogs had leukopenia. All animals showed toxic PMNs with positive bacterial culture. None of the cases in the study showed any quantitative alterations in PMNs such as: neutropenia or neutrophilia, where 100% had nuclear displacement of the regenerative type of PMNs to the left. 100% cases had toxic morphological changes: 90.0% had PMNs with toxic granulations, 80.0% had giant rod neutrophils, 70.0% had target PMNs, in 50.0% of those with vacuolation in the cytoplasm, in 40.0% of the animals, the presence of giant PMNs, 10.0% with Döhle bodies, and another animal 10.0% with karyorrhexis. All case studies had at least one association of two types of toxic changes. Toxic morphological alterations observed in PMNs through cystoscopy proved to be more reliable and sensitive in evidencing the diagnosis of infections than the quantitative alterations of absolute values of total leukocytes; therefore, they were essential in the laboratory diagnosis by blood count in the course of infections in dogs.

## Resumo

Os neutrófilos (PMNs) são marcadores celulares para o diagnóstico de inflamação e/ou infecções. Neste estudo, o objetivo foi apresentar a importância das alterações morfológicas tóxicas dos PMNs envolvidos como marcadores de infecção, em 10 casos com isolamento bacteriano positivo por cultura devido à disúria, hematúria e/ou urina fétida, como manifestações clínicas de doença do trato urinário. As observações de esfregaço foram realizadas para contagens e avaliações morfológicas de 3.000 leucócitos, em esfregaços e em concentrado de leucócitos. Um (10,0%) dos cães teve leucocitose e dois (20,0%) cães apresentaram leucopenia. Todos os animais tiveram alterações tóxicas em PMNs (100% dos casos) e com cultura bacteriana positiva. Nenhum dos casos do estudo apresentou qualquer alteração quantitativa nos PMNs como: neutropenia ou neutrofilia onde 10 (100%) tiveram deslocamento nuclear do tipo regenerativo de PMNs para a esquerda. 10 (100%) casos apresentaram alterações morfológicas tóxicas: nove (90,0%) tinham PMNs com granulações tóxicas, oito (80,0%) tinham neutrófilos de bastonetes gigantes, sete (70,0%) tinham PMNs-alvo, em cinco (50,0%) daqueles com vacuolização no citoplasma, em quatro (40,0%) dos animais a presença de PMNs gigantes, um (10,0%) com corpos de Döhle e outro animal (10,0%) com cariorrexia. Todos os casos observados tiveram pelo menos uma associação de dois tipos de alterações tóxicas. As alterações morfológicas tóxicas, observadas nos PMNs através da cistoscopia, foram mais confiáveis, e sensíveis em evidenciar o diagnóstico de infecções, do que as alterações dos valores absolutos de leucócitos totais e mesmo de PMNs em exames de rotina no curso de infecções em cães.

## Introduction

Leukocytes are cellular biomarkers in the diagnosis of inflammation and/or infections, (Banga et al., 2020; Chmurska-Gąsowska et al., 2021; Gori et al., 2021; Hampson et al., 2017; Ng et al., 2019; Paolino & Williams, 2021; Papasouliotis & Murphy, 2021; Thrall et al., 2012; Vidya et al., 2021). Among the inflammatory response cells, neutrophils (PMNs) are the most numerous leukocytes in dogs, being the main cell population in the inflammatory/infectious response (Burn et al., 2021; Goggs et al., 2020; Ng et al., 2019; Oliveira-Costa et al., 2022), and due to the action of chemotactic substances, produced by cellular or molecular mediators, they migrate chemotactically to the sites adjacent to the inflammation site, where they are able to develop phagocytic and microbicidal activities, thus decreasing, the toxic and morbid effects of pathogenic microorganisms (Hidalgo et al., 2019; Mayadas et al., 2014; Ng et al., 2019; Oliveira-Costa et al., 2022; Overbeeke et al., 2022; Papasouliotis & Murphy, 2021; Yipp et al., 2017). Infections and inflammation produce an acute response, which accelerates the process of neutrophilopoiesis, stimulating the release of PMNs, resulting in the mobilization of large amounts of mature, immature, and even “toxic” morphological changes. As for toxic PMNs, of the type with granulations, they correspond to the accumulation of the microbicidal enzyme myeloperoxidase in the phase before medullary maturation; they are usually associated with quantitative and morphological changes, observed in the leukogram/hemogram, evidenced by a specific relative and/or absolute count, by microscopy. Thus indicating the intensity and reactivity not only by counts, but mainly according to the degree of neutrophils with granulations, being proportional to infection/inflammation (Bastos et al., 2016; Burton et al., 2013; Lilliehöök et al., 2016; Oliveira-Costa et al., 2022; Polton, 2013; Thrall et al., 2012; Urrechaga et al., 2018). These changes observed in the study of these cells in cystoscopy allow us to confirm, diagnose, monitor and quantify the severity of infectious and Inflammatory processes; because in general, serious infections can be observed in the consistent presence of leukocytosis with neutrophilia or not, and nevertheless, morphological changes in neutrophils, mainly toxic granulations, Döhle bodies, toxic vacuolizations, target neutrophils and karyorrhexis, were always present in patients with bacterial infections associated with changes in circulating immature granulocytes according to the increased risk of death (Bastos et al., 2016; Lilliehöök et al., 2016; Papasouliotis & Murphy, 2021; Polton, 2013; Thrall et al., 2012; Vidya et al., 2021; Weiss & Wardrop, 2011), as the potential for bacterial toxemia reflects a medullary effect on neutrophilopoiesis with increased nucleic acid content in the cytoplasm of PMNs in response to bacterial infections (Oliveira-Costa et al., 2022; Urrechaga et al., 2018). The study of neutrophils and “toxic changes”, a term used for the morphological changes associated with infections or inflammatory disorders (Gossett et al., 1985), bringing the importance of the neutrophil phenotype to a new focus of research on the pathology of infections and sepsis, which would potentially make the diagnosis and monitoring of these pathologies more reliable (Ellett et al., 2018; Paolino & Williams, 2021; Troìa et al., 2017), thus seeking a more sensitive examination and the recognition of any morphological variations and, therefore, evidence of functional status, in response to stimuli such as infections (Mayadas et al., 2014; Paolino & Williams, 2021; Urrechaga et al., 2018). These assessments of morphological changes in neutrophils have been extremely important for the prognosis of infection in the animal; since, in most cases, these changes precede the clinical manifestations, allowing an early diagnosis of infections (Aroch et al., 2005; Inkelmann et al., 2012). The toxic effects during granulopoiesis have been reflected in the observations of the microscopic report of hemograms, where cytoplasmic basophilia, presence of toxic granules, vacuoles, Döhle corpuscles, nucleus with abnormal segmentation and the production of giant and bizarre PMNs are observed (Bastos et al., 2016; Lambert et al., 2016; Paolino & Williams, 2021), being released before maturation in the bone marrow, due to stimulation of neutrophil poiesis in a severe infection, where the amount and type of toxicity were related to the severity of the infection disease (Lambert et al., 2016; Paolino & Williams, 2021).

Endotoxic shock induces greater vacuolization of PMNs compared to more benign conditions. As well as the vacuolization of PMNs, and have a direct correlation with serum lactate, a known marker of severe shock, not always requested (Campos et al., 2017). Due to the rapid renewal of PMNs, rapid and marked changes in cell count can occur, observed in serial blood counts, obtained at intervals of a few hours due to the dynamics of leukokinetics. On the other hand, the number of toxic neutrophils would be a useful prognosis (Bastos et al., 2016; Bau-Gaudreault & Grimes, 2019; Polton, 2013; Thrall et al., 2012), especially in infections in dogs, such as those in urinary tract (UTI), which can lead to sepsis from an infectious focus. This is due to the lack of early diagnosis, which could be explained by the absence of specific clinical and laboratory manifestations, which makes it difficult to determine the appropriate treatment and, often, the absence of a diagnosis.

The present work aims to report the importance of morphological changes of neutrophils in the diagnosis of bacterial infections in systemic blood samples, observed in 10 case reports in dogs with infection and positive culture for pathogenic microorganisms with a clinical diagnosis of urethritis.

## Material and methods

### Study area

The report of 10 cases of blood samples from adult dogs, nine males and one female, with suspected lower urinary tract disease, where clinical signs consisted of dysuria, hematuria and/or foul-smelling urine was analyzed.

### Study subjects

The data used were based on results of clinical and complementary laboratory evaluations obtained from patients' medical records. Ten dogs presenting with clinical signs compatible with urinary tract disease - UTI and exhibiting one or more of the following alterations: abdominal palpation pain, hematuria, dysuria, and polyuria, or with a pre-existing clinical history of these conditions were attended to. Urine samples were obtained by cystocentesis after preoperative antisepsis of the abdominal region with sterile disposable material and analyzed using the standard urinalysis examination for physical-chemical evaluations, with urinary density assessed using a manual clinical refractometer^a^. The urinary parameters used to characterize and classify the previous clinical suspicion of urinary tract infection in the selection of dogs were: present and concomitant proteinuria and pyuria, according to a colorimetric reaction^b^ using the cross-referencing to evaluate the intensity (+ to ++++), supplemented by microscopic observations of the sediment after centrifugation of 5 mL of urine (1,500 RPM for 10 minutes) in a centrifuge^c^, where pyuria was observed using a 40X objective^d^, as observed by Thrall et al. (2012).

Blood samples were processed in the Veterinary Service itself using an automatic device^e^, and microhematocrit centrifugation^f^ was used to prepare hematocrits, with subsequent determination of total protein by breaking and using plasma from the microhematocrit tube via a manual clinical refractometer^b^. In addition, the methodology used for the hemogram included the preparation of four blood smears immediately after sampling, without contact with EDTA, according to Lv et al. (2015) and Bastos et al. (2016), which were stained with a rapid differential staining kit in hematology^g^ and observed by optic microscopy^c^, with a specific count of 1,500 leukocytes. The number of observed cells was converted from relative values (%) to absolute values (/µL) in the reports, with observations of possible changes and morphological evaluations of blood cell elements, with particular attention to phenotypic changes in PMNs. Smears with leukocyte and platelet concentrates were prepared using the same fixation, staining, and observation techniques as the blood smears to complement the morphological evaluations.

The urine was immediately cultured after collection according to Agência Nacional de Vigilância Sanitária (2014), Howard et al. (2021) and, Sørensen et al. (2016), where bacterial growth was evaluated using culture media, and information was complemented by performing antibiograms and susceptibility tests using the Kirby-Bauer disk diffusion method^h^ in accordance with the Clinical Laboratory Standards Institute (CLSI) guidelines (Agência Nacional de Vigilância Sanitária, 2014; Oplustil et al., 2010). The microorganisms were identified by qualitative culture methodology. The sample was seeded by the plate depletion technique on Petri dishes containing MacConkey^i^ agar and 5% sheep blood agar. In addition, the materials from the 10 samples were inoculated into Thioglycolate^j^ broth for control and recovery of microorganisms incubated in a microbiological oven at a temperature of 35±1°C for 48 hours, with growth analysis at 42°C for control.

## Results

Ten dogs included in the study had clinical signs with concurrent presence of pyuria and proteinuria, with varying degrees of intensity (+ to ++++), and positive urine culture of a sample obtained by cystocentesis, confirming the diagnosis of urinary tract infection. All ten dogs presented toxic morphological changes of neutrophils in microscopic evaluations, regardless of the microorganisms isolated in urine cultures (Table 1). 50% of the isolated microorganisms were associated with *Escherichia coli*, 20% with *Klebsiella pneumoniae*, and 10% with each of the etiological agents identified as *Staphylococcus aureus*, *Enterococcus faecalis*, and *Trichosporon* spp., respectively.

**Table 1:** Number and classification of toxic neutrophils observed in blood samples from dogs with urine isolates associated with different etiological agents.

Microbial isolates		Neutrophil morphologies							Changes:	Diagnosis*	
		Toxic granulations	Vacuoles	Target	Karyorrhexis	Döhle’s Corpuscles	Giants	Giants rods		Leukopenia	Leukocytosis	
1.	*Escherichia coli*	5,740	1,148	-	-	-	-	-	2	**7,000**	**--**	
2.	*E. coli*	11,840	237	-	-	-	-	-	2	**-**	**18,500**	
3.	*E. coli*	-	140	140	-	-	-	53	3	**5,300**	**-**	
4.	*E. coli*	7,047	-	282	-	-	-	2,282	2	**-**	**-**	
5.	*E. coli*	1,000	1,100	200	-	-	300	2,200	5	**-**	**-**	
6.	*Klebsiella pneumoniae*	10,920	-	-	-	-	-	140	2	**-**		
7.	*K. pneumoniae*	-	-	213	53	-	106	76	4	**-**	**-**	
8.	*Staphylococcus aureus*	4,332	-	87	-	-	-	-	2	**-**	**-**	
9.	*Enterococcus faecalis*	7,020	-	-	-	-	211	701	3	**-**	**-**	
10.	*Trichosporon* spp.	11,049	-	110	-	110	-	1,127	4	**-**	**-**	

* According to Thrall et al. (2012). Observations: 50% with *Escherichia coli*; 20% with *Klebsiella pneumoniae*, 30% with other etiologic agents.

**Figure 1:**
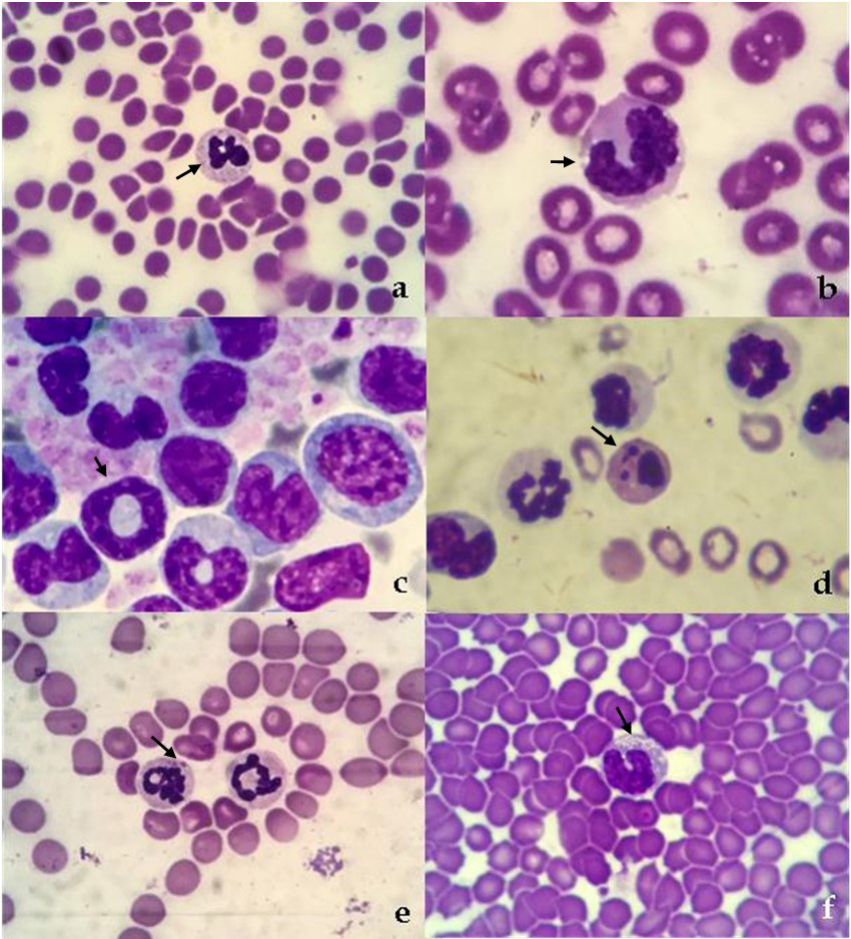
Photomicrograph of toxic neutrophils in dogs with urinary tract infection clinical signals (→) in blood smears: (a) Granulations; (b) Vacuolization and giant; (c) Target form; (d) Karyorrhexis; (e) Döhle bodies; (f) Rod form. Fast Panoptic. Obj. 100X.

The number of neutrophils observed was described in reports, from relative values (%) and transformed into absolute values (/µL). One of the most common toxic changes was the presence of small structures in the cytoplasm in the form of tiny azurophilic granules or reddish-pink staining in the cytoplasm of PMNs in 80% of the cases observed here, regardless of the species of microorganism isolated (Figure 1a). Another toxic change found was the presence of cytoplasmic vacuoles or cytoplasmic vacuolization (Figure 1b) in 50.0% of the cases evaluated here. In addition to this change, giant PMNs were observed in 30.0% of the animals (Figure 1b), which differ from giant rods by not having a segmented nucleus (Figure 1f) and can be seen alone or in more than one cellular alteration. In seven of the 10 animals (70.0%), the nuclei were larger than normal (Figure 1f). In addition to this change, target or ring PMNs can be observed (Figure 1c) in six of the 10 animals (60.0%) that presented the respective alteration; these are cells that have a ring-shaped nucleus.

Karyorrhexis or apoptosis (Figure 1d) was observed in only one of the animals (10.0%). This change indicates the importance of studying apoptosis or the presence of karyorrhexis or neutrophils with cellular death, including as confirmation of organic aggression by infection and/or inflammation.

Döhle bodies (Figure 1e) were observed in one of the animals, characterized as grayish inclusions in the cytoplasm that result from the lamellar aggregation of the Rough Endoplasmic Reticulum (RER), also indicating a systemic inflammatory and/or infectious process, as observed in the animals of this study.

## Discussion

There is a close direct positive relationship with other clinical and laboratory markers, confirmed by the isolation of microorganisms (Campos et al., 2017; Oliveira-Costa et al., 2022; Paolino & Williams, 2021). Parts of this process include total and specific leukocyte counts, calculation of the absolute value per mL of blood, and morphological examination of leukocytes on the stained blood smear.

This reported information makes up the WBC, absolute cell numbers, rather than relative percentages, which can be misleading, should be used in interpreting the WBC response, and the individually specified WBC types should be determined, with the greatest number of cells counted and evaluated for a more reliable result, as the observation of direct stretches and smears of leukocyte concentrates from systemic blood has been useful to clarify the diagnosis and contribute to appropriate treatment (Bastos et al., 2016; Lv et al., 2015; Papasouliotis & Murphy, 2021). On the other hand, the use of automation through hematology counters has not added any additional benefit, when used in a unique way (Troìa et al., 2017), or provide quantitative information without complementary on the morphological characteristics of PMNs, monocytes and lymphocytes; thus not allowing, with the morphological evaluation, a more detailed examination and showing the need to detect which morphological variations, and therefore functional *status*, in response to stimuli, as in infections (Oliveira-Costa et al., 2022; Paolino & Williams, 2021; Zhang et al., 2021), therefore, the special attention of neutrophils is necessary, as it is confirmed that the study of neutrophils presents an accuracy in the diagnosis of infection and sepsis greater than 98%, as observed at the present study, which brings the observation of neutrophilic changes in 100% of patients, as another important diagnostic parameter, since the neutrophil phenotype can potentially diagnose, monitor infection and sepsis (Anderson & Singh, 2018; Ellett et al., 2018; Kim et al., 2009; Oliveira-Costa et al., 2022; Zhang et al., 2021) confirming the relationship between the presence of toxic alterations and microbiological infections confirmed by the isolation results (Paolino & Williams, 2021; Salgado et al., 2007) as observed in the 10 case reports in the present study.

The observation of toxic granulations, in neutrophils, and reflect the infection state of the organism, helping in the early diagnosis of infection (Ayres et al., 2019; Buoro et al., 2018; Paolino & Williams, 2021; Zhang et al., 2021). Nine out of 10 animals (90.0%) presented this abnormality (Table 1), toxic granulations are small structures observed in the form of azurophilic granules or red-pink colored granules in the cytoplasm of neutrophils, due to the retention of mucopolysaccharide acid, which is normally lose in neutrophil maturation, appear as dispersed, small granules, commonly seen in cases of severe toxemia (Ayres et al., 2019; Bastos et al., 2016; Buoro et al., 2018; Garland, 2011; Paolino & Williams, 2021; Yipp et al., 2017; Zhang et al., 2021) or they may present toxic manifestations through cytoplasmic basophilia, a change that appears in the grayish blue to dark blue coloration of the cytoplasm, present in bacterial infections associated with inflammation (Garland, 2011), opposite to the normal neutrophil cytoplasm, and which can also manifest in young forms, where this basophilia is a result of retention of ribosomes and RER, due to accelerated granulopoiesis (Polton, 2013; Thrall et al., 2012; Weiss & Wardrop, 2011). They may also result from endocytosis of toxic agents, with the formation of abnormal granules (Salgado et al., 2007). The granules contain proteins that limit bacterial proliferation and confirm infection (Al-Gwaiz & Babay, 2007; Oliveira-Costa et al., 2022).

The occurrence of horseshoe shapes is due to abnormal mitotic divisions during the development of neutrophil precursor cells, with spots, which vary from gray to dark blue in the cytoplasm, they have a large nucleus, chromatin aggregation, cytoplasmic basophilia and weak granulation (Aroch et al., 2005; Bastos et al., 2016; Hampson et al., 2017). This occurs as an inflammatory response to infection, where these cells are produced in an accelerated way by leukocytopoietic organs, responding to an endotoxemia, confirming the presence of infection as observed in the present study (Blackwood, 2016; Hampson et al., 2017; Lilliehöök et al., 2016; Liongue et al., 2009; Paolino & Williams, 2021; Polton, 2013; Thrall et al., 2012; Weiss & Wardrop, 2011).

The target forms were the main nuclear alterations typically related to infection and sepsis; they are typical of promyelocytes and become visible also in toxic myelocytes. Donut-shaped nuclei are abnormal in dogs and primates, but are a normal finding in rodents. Dysplastic changes can be viewed as defects in nuclear or cytoplasmic maturation and result from interference with DNA synthesis and include megaloblastosis, large cell size, atypical mitotic figures, fragmented nuclei, and binucleation associated with severe infections (Bastos et al., 2016; Blackwood, 2016; Lilliehöök et al., 2016; Polton, 2013; Thrall et al., 2012; Weiss & Wardrop, 2011).

Neutrophilic degranulation is a common finding in infections directly associated with increased immune response, it is due to degranulation or loss of granules released to fight bacterial proliferation in the infectious focus and confirm infection (Oliveira-Costa et al., 2022; Paolino & Williams, 2021; Santopolo et al., 2021). The granules released when membrane integrity is lost during maturation or, vacuolization can develop as an artifact in stored samples, which did not occur with the present work (Bastos et al., 2016). Vacuoles are clear spaces, 1 to 5 µm (equivalent to 5x10^6^ mm in diameter), which appear at first discretely, leading to a loss of granular uniformity, progressing to an intensification of foamy cytoplasm (Aroch et al., 2005). This may also be a result of severe systemic toxicity and cellular digestion, where bacterial toxins can induce the breakdown of lysosomes, releasing lysozymes (Campos et al., 2017; Harvey, 2017; Polton, 2013; Thrall et al., 2012; Weiss & Wardrop, 2011). Visualization of these vacuoles usually disappears 12 to 24 hours after initiation of treatment (Emerson et al., 1970), vacuolated neutrophils have occurred in severe infections serving as an indicator of severe infections (Jafri & Kass, 1998).

According to Aroch et al. (2005) and Bastos et al. (2016), they are released from the bone marrow or by organs, such as the kidney and spleen, as a result of an inflammatory response to infection, when observed in systemic blood smears, or from washings or effusions, they confirm an infectious and/or inflammatory process (Bastos et al., 2016; Blackwood, 2016; Campos et al., 2017; Harvey, 2017; Liongue et al., 2009; Polton, 2013; Thrall et al., 2012; Weiss & Wardrop, 2011). This is a challenging area of neutrophilic *status*, where bacterial phagocytosis is one of the actions performed by PMNs, which accelerates apoptosis, which ultimately promotes resolution of the infection. However, some bacterial pathogens have competence to produce neutrophil apoptosis, and thus cause disease (Burn et al., 2021; Kennedy & DeLeo, 2009).

Neutrophils have characterized by a lifespan, and the assessment of apoptosis is interesting because many cytokines and substances produced by pathogens can influence the rate at which neutrophils undergo apoptosis/karyorrhexis and/or necrosis. Cells undergoing apoptosis or karyorrhexis appear pyknotic or cariogenic and are found infrequently in freshly collected blood, handled and processed properly, according to the present study. Pyknotic cells in peripheral blood have been reported in humans with inflammatory and neoplastic conditions. In a study, apoptotic leukocytes in the peripheral blood of humans were more common. Their presence was most often associated with infection or neoplasia, and the amount is related to the pathogenicity of the infectious etiologic agent (Bastos et al., 2016; Burn et al., 2021; Kennedy & DeLeo, 2009; Polton, 2013; Thrall et al., 2012; Weiss & Wardrop, 2011; Wilcox & Russell, 2008). The vast majority of Döhle bodies has appeared single, oval or rounded, irregular in shape, and often situated on the periphery of the cell. They are more common observed, mainly in the feline species and in equines than in other animal species. In dogs, it is always present when there is an intense demand for a neutrophilic response (Al-Gwaiz & Babay, 2007; Aroch et al., 2005; Blackwood, 2016; Campos et al., 2017; Garland, 2011; Harvey, 2017; Polton, 2013; Thrall et al., 2012; Weiss & Wardrop, 2011).

The isolation of the etiologic agent as a confirmation of bacterial infection (Table 1) and the relationship with the cellular morphology of PMNs and their respective alterations (Figure 1), with their presence in the hemogram results, show an association between clinical manifestations, presence of toxic alterations and the results of positive cultures, present in 100% of the patients. Thus, our data suggest that even in the absence of leukocytosis, neutrophilia and left shift, they cannot be considered as excluding factors for the presence of infection, but they can still be considered risk factors for a poor prognosis (Al-Gwaiz & Babay, 2007; Banga et al., 2020; Bastos et al., 2016; Burton et al., 2013; Oliveira-Costa et al., 2022; Paolino & Williams, 2021; Papasouliotis & Murphy, 2021; Zhang et al., 2021), as well as the negative culture is not an exclusion factor for infection (Ottolini et al., 2003), especially when the degenerative shift of neutrophils to the left is present, associated with an increased risk of death or euthanasia that, in the however, should be interpreted in conjunction with the diagnosis of the disease and the correlation with the patient (Burton et al., 2013).

Nevertheless, the present evaluation refers to the potential for diagnosis of the infection, since all animals presented toxic and positive culture, confirming that the evaluation of blood smears can provide useful clinical information and can serve for the diagnosis and prognosis of patients (Al-Gwaiz & Babay, 2007; Aroch et al., 2005). All dogs in the study (100%) had at least two types of toxic alteration (Table 1), regardless of the etiological agent diagnosed, in agreement with the association of infections and toxic alterations present (Al-Gwaiz & Babay, 2007; Banga et al., 2020; Bastos et al., 2016; Burton et al., 2014; Harvey, 2017; Oliveira-Costa et al., 2022; Paolino & Williams, 2021; Papasouliotis & Murphy, 2021; Zhang et al., 2021).

The normal leukocyte count in the hemograms, different from that described by Rebar et al. (2003), in which it is not the amount of leukocytes that will more sensitively indicate whether there is indeed an infectious and/or inflammatory process installed or not, but the presence of toxic neutrophils, as was observed in all cases of the present study, with the presence of 100% of some type of toxic change in neutrophils. According to Bastos et al. (2016) and Schultze (2011), toxic neutrophils were always associated with severe infections, accelerating the process of neutrophilopoiesis, with subsequent release of these cells from the marrow before their maturation. Acute inflammation results in the mobilization of large amounts of mature and immature neutrophils, traditionally seen as important effector cells in fighting infections, which theoretically increases the number of these cells in the circulation, absolutely increasing the number of neutrophils, with immediate migration to the affected organ, producing a leukocytosis by neutrophilia (Pillay et al., 2012; Yipp et al., 2017), a result observed in a single patient, however, the microscopic observation of blood smears that detected toxic changes of neutrophils and showed it is valuable in 100% of patients (Bastos et al., 2016; Lambert et al., 2016).

In general, bacterial infections should be observed in the presence of leukocytosis with neutrophilia, as well as morphological changes in neutrophils, such as toxic granulations, Döhle corpuscles, and toxic vacuolizations (Chang et al., 2017).

The absolute numbers that constitute the normal values for neutrophils are controversial and may be dependent on age, comorbidities, and organ affected, as already demonstrated using an established flow cytometry method to characterize the anatomical location of organ-specific neutrophils (Yipp et al., 2017). There are several diseases and drugs that produce variations in leukocyte values (Blackwood, 2016; Kritsepi-Konstantinou & Oikonomidis, 2016), agreeing that the visualization of toxic changes are important parameters in the diagnosis of infections, their determination and intensity inform a clinical and allows the therapeutic intervention necessary for proper patient management, its observations confirm infections and are correlated with microbiologically proven infections (Bastos et al., 2016; Hampson et al., 2017; Mayadas et al., 2014; Oliveira-Costa et al., 2022; Paolino & Williams, 2021; Zhang et al., 2021), observed in the present reports.

## Conclusions

Neutrophil cytopathology can diagnose infections in dogs. The presence of toxic neutrophils indicates the presence of infections. The greater number of toxic cells observed, the higher the pathogenicity of the bacterial infection and has a greater chance of undergoing to sepsis. The absence of leukocytosis, neutrophilia, and rod neutrophils does not necessarily mean the absence of bacterial infection.

## Footnotes

a Modelo Q667 (Quimis Aparelhos Científicos).

b Fita Combur-Test®Roche Diagnóstica Brasil Ltda.

c Mod. 208N, Excelsa Baby, marca Fanem Ltda.

d Eclipse E200, Nikon Instruments Inc., Japão.

e Ms4-Vet-Melet Schloesing Lab. Couter.

f E3500108 Microspin CDR.

g Fast Panoptic LB- Laborclin prod. para lab., Ltda, Pinhais, PR.

h IMUNOVA análises biológicas, Curitiba, PR.

i Kasvi® São José do Pinhais, PR ^j^MicroMed®

j LABORCHEMIKER Curitiba, PR.
